# Burns and Scalds

**Published:** 1885-05

**Authors:** J. McF. Gaston

**Affiliations:** Atlanta, Ga., Professor of the Principles and Practice of Surgery


### BURNS AND SCALDS.

A CLINICAL LECTURE DELIVERED AT THE SOUTHERN MEDICAL COLLEGE, BY J. MCF. GASTON, M. D., ATLANTA, GA., PROFESSOR OF THE PRINCIPLES AND PRACTICE OF SURGERY.

Reported for the ATLANTA MEDICAL AND SURGICAL JOURNAL.

GENTLEMEN: The frequency of the injuries from burns and scalds warrants some special remarks touching this class of cases, based upon the large extent of surface involved in the patient presented for your examination to-day, connected with another which I have been called to treat in consultation recently.

This mulatto woman, about 25 years old, reports that she was burnt on the 17th of January, while sitting asleep in front of a fire, and that she was without any kind of treatment for twenty-four hours afterwards. She was then seen by one of the city physicians, who applied a mixture of linseed oil and lime water; and for two weeks she was not seen again, but continued this local application to all the injured surfaces. Another physician was then called to see her, who doubtless proceeded properly in continuing this course.

She entered the hospital here on February 11th, when, upon examination of the extensive ulceration over the entire left side of the thorax, the left arm, and the left thigh, the mixture of equal parts of linseed oil and lime was still thought to be the most suitable application. As you observe, the left mammary gland is involved, and she states that this was the only part from which the outer layer of the skin was detached upon changing her clothing after receiving the burn. It is a matter of surprise that no more constitutional trouble ensued than is stated by the patient, and that no greater pain should have followed the injury within the first day. The insensibility to suffering might be attributed to the depression of the vital forces, but there is no history of such general prostration in the case, and yet the deep-seated implication of the cutaneous structure is clear from the protracted ulceration which is present still, after a month has elapsed.

In view of the persistence of the inflamed condition under the use of this combination, from the outset to this time, it might be supposed that the remedy had not served a good purpose, were it not that in many similar conditions the most happy effects have attended its use, in my own experience and in the hands of others. This mixture of linseed oil and lime water is canonized as the best thing for superficial burns, such as we have in this case, and yet its healing properties seem not to have secured the desired result, so that now I shall undertake to stimulate the callous ulceration by an application of a different kind that has been used with advantage under such circumstances. A combination of three ounces of sweet oil, one ounce of spirits of turpentine, and one drachm of camphor, will therefore be now applied continuously upon fine cloths next to the ulcerated surface, which may remain for some days, while other cloths soaked in this mixture may be placed over them from time to time. The object in retaining the cloths placed next to the surface is, in part, to prevent the exposure to the contact of the atmosphere, but also to obviate the irritation of the denuded tissues from frequent changes of the dressing, and I would commend this especially to your attention.

As correlated with this treatment, I may state that after resorting to it, in another case to which I have already referred, the indolent inflammation of the surface of the burn upon the thighs of a little girl was soon transformed into exuberant granulations, with suppuration. With a view to modify this hyperæmic condition, a slightly astringent application was indicated, and my colleague concurred in the use of Goulard's cerate, spread upon fine cloth, to the entire granulating surfaces. This mild salve has exerted a most favorable influence, and the healing process is proceeding satisfactorily.

In the case before us a similar salutary effect may be expected after a change has been effected by the stimulating liniment now prescribed, and I hope, after a short time, to resort to the Goulard's cerate with advantage.

I have thus briefly detailed the measures upon which reliance is to be placed in ordinary cases of superficial injury from burns. But our resources are not by any means limited to these means of cure, and as you will have to meet the exigencies of a great variety of cases presented in practice, a few practical hints may be given for your guidance in the lesser and grosser phenomena of recent and long-standing injury of the several textures which may be involved. It may not be irrelevant to state that the several modes in which the tissues are affected by a high temperature vary according to the intensity of the heat and the duration of contact with the part. There may be an aggravation of the injury inflicted by the force of an explosion in which either dry heat or steam impinges violently against the surfaces, and the extent of the mischief depends upon the character of the agent.

The division of burns, by the French, into six varieties, has been followed, to some extent, in the descriptions of English and American authors, but for practical purposes it is preferable to adopt the classification of Ziegler into three degrees of severity: 1st. Merely erythematous reddening of the skin. 2d. The formation of blisters. 3d. The destruction of some portion of the fibrous structures.

In the annotations upon the article on burns and scalds, in Holmes' Surgery, Dr. Morton indicates this triple division as the simplest, qualifying the superficial skin irritation as hyperæmic ; the proper cutaneous inflammation being that in which vesicles are formed ; and the disorganization of the true skin, or deeper seated parts, are comprehended under the heading of eschars, extending to greater or less depth in the tissues. The slightest impression made upon the cuticle by heat, from any source, is attended with mere superficial redness, with a stinging sensation of the surface, and tenderness to the touch. If confined to a small area, there are no serious consequences, but if such superficial burns spread over a large extent of the skin, they interfere so materially with the functions of this investment of the body as to assume a grave character. There is a notable depression of the vital powers from the large extension of an apparently very slight impression of a flame or other means of bringing heat suddenly in contact with the surface. This partakes of the nature of shock through the nervous system, and doubtless depends upon that intimate relation of the minute terminations of the nerves and the capillaries with the nerve centres, which, you will observe, that I have indicated in the essay placed in your hands at the opening of the session. By a careful study of it, you may find a solution of the remarkable fact that cases of epilepsy have been averted by burns of the extremities. In view of my "explanation of the pathology and therapeutics of the diseases of the nerve centres," it is evident that the nerve centres are essential for the propagation of any impression upon the superficial distribution of nerves.

For a satisfactory comprehension of the condition of the nerves and capillaries, and their relations to the nerve centres, we must look to a property of the nervous system, which may be defined as the excito-dynemic element, and it is this vitalizing function which is impaired in extensive superficial burns. They are then to be viewed always as very grave accidents, from the depressing influence which is exerted upon the brain and heart. There may be only a general redturgence of the surface, without vesication in any part, from a comparatively slight local contact of steam or flame over a large portion of the body, and yet prostration of the vital powers may ensue, owing to the impairment of that nervous development which connects the surface with the interior structures of the body. Burns or scalds, therefore, of the abdominal parietes, or the outer walls of the thorax, as in the case before us to-day, must be regarded with great circumspection, and the general condition of the system closely watched, not only at the outset, but in the further progress of the case. I cannot agree with Mr. Morris when he states, in the last volume of the *International Encyclopedia of Surgery*, that the constitutional symptoms, prognosis and treatment of such injuries do not differ from those of burns and scalds of other parts of the body. The sympathies of the surface with the internal viscera in such cases certainly has an importance and gravity greater than in burns of corresponding degrees in other regions, his statement to the contrary notwithstanding.

We learn from the observation of cases, and from writers on this subject, that when the skin is exposed to the action of heat in such a way that the epidermis is killed, while the underlying cutis and its vessels are injured, but not killed outright, blisters are formed on the surface; in other words, the skin, reddened by congestive hyperæmia, is raised into vesicles or blots containing clear liquid.

This constitutes the second degree in this triple classification, and it is also in that division made by authors into six classes; while the third degree of Ziegler includes all the stages of severity comprised by Dupuytren in his third, fourth, fifth and sixth degrees. Ziegler states in his Pathological Anatomy that in burns of the second degree it often happens that not all of the epidermal layers become necrotic, but only the surface layers. The general course of the process and the accompanying series of texture changes will accordingly vary in different cases.

Usually the last epidermis is quickly replaced by regenerative growth under cover of the horny shell of the blister. The exuded liquid disappears by evaporation, and if, after a day or two, the dry shell of the epidermis be removed, the denuded surface will be found completely covered with new epidermis, and the injury is indicated only by the bright red tint of the spot.

When a blister containing a serous effusion is evacuated by puncture at an early period, and the cuticle is brought into contact with the true skin and kept approximated by gentle pressure, union is generally effected and completely re-established without any secondary inflammation.

But Ziegler tells us that when irritant matters gain access to the denuded papillary surface, as may happen if the skin of the blister is prematurely removed, the healing process is notably delayed. The exudation of liquid and cells continues for some time longer, the deep-red surface secreting a more or less tinted liquid as catarrhal exudation. But presently this ceases and the epidermis is reproduced. This new protracted process of repair is to be looked for when the injury caused by the burn extends to the papillæ and deeper layers of the cutis.

In the third and final degree of burns, as given by Ziegler and Morton, the eschar may involve not only the proper textures of the true skin, but extend to the tissues beneath. It has been held as a diagnostic of the implication in the injury of the whole skin, that the sensitiveness upon pressure is much less than when the burn does not involve all its structure; and that deformity is much more likely to ensue in the former than in the latter condition.

Contraction in healing of burns seems much more frequent than in other forms of ulceration of the same extent, and when the muscular, as well as the sub-cutaneous cellular tissue suffers, deformity is the ordinary consequence. All are familiar with the hideous distortions of the features which occur from the contracted cicatrization of burns about the face and neck, and with the serious disabilities produced by the irregularities in the muscles and tendons of the upper and lower extremities from the effects of burns on these parts.

The remedial operations which have been resorted to by surgeons have not been attended with the most satisfactory results, and yet, in cases where the indurated structures can be completely dissected away, and their place supplied by plastic process upon the surrounding sound tissues, operations give promise of success.

The general indications for the local treatment of burns and scalds correspond, except in those aggravated injuries resulting from the direct contact of fire with the tissues, consisting in carbonization of the structure. In such destruction of the texture and vitality of a part, sloughing must necessarily result, and has to be managed upon the same principles as eschars from other causes.

In the slighter effects of heat upon the surface, such as occurs from protracted exposure of the naked body to the action of the sun, or from the effusion of hot water upon the skin, there is frequently a diffused inflammation, requiring soothing applications, and a great variety of means have been used in domestic practice. For ordinary burns, cold water at the outset is the allopathic remedy; whereas, holding the part to the fire continuously does its work upon the homeopathic doctrine, that like cures like. The following local remedies are useful: Lard with flour, and covered with cotton batting, bicarbonate of soda with lard, camphor with sweet oil, spirits of turpentine, lye soap, honey and starch, carbonate of lead with oil, in the form of white paint, and the best of all, after the first dressing, is perhaps the mixture of linseed oil and lime water, of which I have already spoken. In all cases the exclusion of the atmosphere from the surface is essential to a good result, and in small, circumscribed, superficial burns, coating the skin immediately with collodion prevents the development of blebs.

The internal remedies required in the constitutional disturbance following extensive burns call for our most serious attention. Opiates, to relieve pain, and at the same time arrest the shock to the nervous system, are of the highest importance at the outset. Subsequently, stimulants, such as carbonate of ammonia, sulphuric ether and musk are indicated to obviate the collapse of the system.

In the consequent suppuration, tonics of Peruvian bark, elixir of vitriol, with milk punch and nutritious diet, are requisite.

